# Injurious Effects of Vulcanized Rubber

**Published:** 1881-01

**Authors:** L. P. Haskell

**Affiliations:** Chicago


### Article IV.

### Injurious Effects of Vulcanized Rubber. By L. P. Haskell.

Sixteen years ago Dr. W. B. Roberts, of New York, wrote as follows, in an article for the *People's Dental Journal*, published in this city:

"We discovered that, of the *red vulcanite*, commonly used by dentists, no less than *one-third consisted of the coloring matter, bisulphuret of mercury*, or the vermillion of commerce; and that the balance was composed of sulphur and rubber, in nearly equal parts. Medical testimony informed us that the bisulphuret of mercury produces the same effect upon the system as pure mercury; we also found that in the process of mixing these ingredients, none of their properties are lost; and by actual experiment, before and after vulcanizing, the weight of the substance is the same.

"It therefore became evident to us that the bisulphuret of mercury and sulphur—two substances confessedly injurious to health—were actual ingredients of the material when placed in the mouth; and common sense, as well as science, taught us that such effects as were perceptible in such mouths, could proceed from no other cause.

"These effects, as noted in our own experience, and declared by the evidence of many others, were, first, *a great absorption of the alveolar and bony substance of the jaw;* next, irritation of the mucous membrane, and *a condition nearly approaching salivation;* then gastric irritation, and, finally, the general derangement of the health consequent upon such abnormal conditions.

"*All of these symptoms of disease were removed by simply removing their obvious cause*, RED VULCANITE; while a healthy condition continued after replacing it with gold, or continuous gums.

"One cause of these inflammatory conditions under red, or any color of vulcanite, is the fact that it is a non-conductor of heat, and this cause, as will be apparent to any physiologist, is sufficient to produce the worst results."

To this testimony of Dr. Roberts has been added sixteen years of experience all over the world—we may say universally—of the dental profession. Doubtless there are many dentists who are unaware of these facts in relation to vulcanite; especially among that class who commenced practice since its introduction, and have had little or no experience in the use of *metal* plates; by many such, these abnormal conditions of the mouth are supposed to be owing simply to the presence of plates, no matter what the material; or, perhaps, owing to some peculiar condition of the system. There are others who ought to know better, that insist there are no injurious effects arising from the use of such materials, and still persist in recommending it as good, if not better than any other material used.

It will be observed that there are two serious objections to the use of these materials, viz.: loss of bony substance from undue absorption, caused by the retention of heat under the plate; and the *poisonous* effects of the coloring material, which constitutes *one-third* of the whole plate.

The first of these is the least serious of the two, but affects all who wear it, only in different degrees. I never have yet seen a mouth where this material has been worn but there were evidences of undue absorption, and thousands of mouths are ruined by it, for absorption goes on until there is no "process" left, and no ridge, or only a flexible one of thickened membrane.

To a young, or middle-aged person, doomed to wear artificial teeth the balance of their days, it is certainly desirable that as permanent a condition of the *ridge* should be maintained as possible.

There is undue absorption under metal plates, sometimes, but, owing, most always, to undue pressure, long continued, upon one portion of the jaw.

The effect produced by the coloring material is far more serious, although not so often realized, because it seriously affects the health of the patient. Dr. Roberts has made so clear a statement, that it is unnecessary for me to add anything but to say that it is corroborated by many members of the profession, during these years past, and also to give several cases, that have come under my own observation.

One year ago, a German, about sixty years of age, was sent to me by Dr. Justin Hayes, whom he had consulted for treatment, and who told him it would be useless for him to do anything until he rid his mouth of a full set of rubber plates, which he had worn for eight years, and during the whole time had suffered in a peculiar manner, as follows: although having a good appetite, yet, the sight of food of whatever nature, with the exception of *fluids*, produced a feeling of disgust, almost horror, so that he subsisted almost entirely upon soups. I advised the use of "Continuous Gum Work," but he said the price was beyond his reach. I told him that perhaps celluloid would remedy the difficulty, and tried it. In two weeks he returned and said the trouble was as bad as ever, after wearing the plates one week, but at Dr. Hayes' suggestion, had left all plates out of his mouth for one week, and he began to improve. I arranged to make him the "Continuous Gum," inserted the plates, and, seeing him ten days after, and not since, until last week (one year), when he told me he had been entirely free from all unpleasant symptoms of the eight years previous.

In this case, I said, I tried the celluloid. This material has the same non-conducting quality of rubber, and in this respect is just as objectionable, but although it has the same coloring matter, there is very much less of it, and I never before observed any such results from its use.

Last March, a lady from Topeka, Kansas, called. For twelve years she had been wearing a rubber plate, and during the entire time had suffered with a serious stomach difficulty, and also a chronic diarrhœa. She had observed the inflamed condition of her mouth, and had queried whether the plate caused it and the stomach difficulty. I made her a set of "Continuous Gum," and she returned home. In about six weeks she accidentally broke off a tooth and sent it to me for repairs, and for a week wore the rubber plate again. During the summer, on her way East, she called and told me her her experience, as follows:

After wearing her new set the symptoms soon began to disappear, and by the time she broke it, considered herself well; but, on putting in the old rubber set, the old symptoms reappeared, and continued to increase until she received her new set again, upon wearing which, the old symptoms again disappeared, and she was well again.

On telling her physician of what she had been doing, he said he did not know she was wearing a rubber plate, and if he had he did not know there was anything injurious in it, but he had been treating her for symptoms of *mercurial poisoning*, but of course without avail.

I could relate other cases of a similar nature, and so could dentists all over the country; but these suffice to illustrate the necessity of a more careful exploration of the mouth to find the cause of symptoms that sometimes puzzle the medical practitioner.

CHICAGO, December, 1880.

